# Diagnostic Approach to IgG4-Related Retroperitoneal Fibrosis After Colorectal Cancer Surgery in a Patient With Normal IgG4 Levels: A Case Report

**DOI:** 10.7759/cureus.63894

**Published:** 2024-07-05

**Authors:** Eiichi Kakehi, Makoto Matsumoto, Kae Sugiyama, Haruka Okutani, Kazuhiko Kotani

**Affiliations:** 1 Department of General Medicine, Tottori Municipal Hospital, Tottori, JPN; 2 Department of Surgery, Tottori Municipal Hospital, Tottori, JPN; 3 Division of Community and Family Medicine, Jichi Medical University, Shimotsuke, JPN

**Keywords:** positron emission tomography, soluble interleukin-2 receptor, retroperitoneal fibrosis, colorectal cancer, igg4-related disease

## Abstract

An asymptomatic 75-year-old man who underwent transverse colon cancer surgery two years previously presented with retroperitoneal fibrosis (RPF) around the ventral sacral and right external iliac artery and vein on abdominal computed tomography (CT) during a routine surveillance visit. We assumed cancer recurrence or immunoglobulin G4 (IgG4)-related disease (RD), but although generic tumor markers and IgG4 levels were normal, soluble interleukin 2 receptor (sIL-2R) was elevated at 569 U/mL (reference: 122-496 U/mL). No diagnosis was made at this time, and the patient was followed up. He subsequently developed edema of both lower extremities. Abdominal enhanced CT showed an enlarged RPF without invasion of surrounding organs and with a delayed contrast effect, and positron emission tomography-CT showed fluorodeoxyglucose accumulation in the same area but a lower standardized uptake value (SUV) than at the time of transverse colon cancer diagnosis. Although generic tumor markers and IgG4 levels remained within the reference range, sIL-2R was further elevated to 1100 U/mL. An open biopsy and histopathology showed a high IgG4/IgG-positive cell ratio and infiltration of IgG4-positive plasma cells. The patient was finally diagnosed with IgG4-RD RPF. In cases of RPF after colorectal cancer surgery, the combined findings of elevated sIL-2R, lack of infiltration into surrounding organs, and lower SUV values ​​than at the cancer site could provide useful information to aid the diagnosis of IgG4-RD RPF.

## Introduction

Immunoglobulin G4-related disease (IgG4-RD) is a systemic autoimmune disease characterized by fibroinflammatory infiltration, which can affect almost all organ systems [1], including the retroperitoneum, leading to retroperitoneal fibrosis (RPF) with IgG4-RD (IgG4-RD RPF) [2]. IgG4-RD RPF is a rare disease in which fibrous inflammatory masses develop around the abdominal aorta, iliac artery, and ureters, as well as retroperitoneal structures. Although RPF is often classified as idiopathic, secondary (e.g., associated with drugs, infection, radiation, and malignancy), and IgG4-RD [3], IgG4-RD RPF has recently been reported following malignancies [4-6].

IgG4-RD is generally characterized by high serum levels of IgG4; however, some patients with IgG4-RD have normal IgG4 levels [1]. In some cases, generic tumor markers do not indicate relapsing cancers, and it is difficult to differentially diagnose IgG4-RD RPF before biopsy in patients with a history of malignancy. Some studies showed that soluble interleukin-2 receptor (sIL-2R) [7,8] or positron emission tomography (PET) computed tomography (CT) [9,10] could aid the diagnosis of IgG4-RD RPF; however, these earlier findings [7-10] are staying in the knowledge, and the combined application of the findings to the clinical diagnosis has rarely been reported. Here, we report on a patient who developed isolated IgG4-RD RPF with normal IgG4 levels after surgery for transverse colon cancer.

## Case presentation

A 75-year-old Japanese man visited our hospital with a chief complaint of edema in both lower legs, especially in the right lower leg. He had a history of surgery for transverse colon cancer (pathological stage IIa) two years earlier and did not receive radiation therapy or chemotherapy either preoperatively or postoperatively. He had experienced a cerebral infarction at 61 years of age and was receiving antiplatelet medication. He also had a history of smoking between the ages of 18 and 48 years and drank “shochu,” providing an alcohol intake of 10 g/day every day.

**Figure 1:**
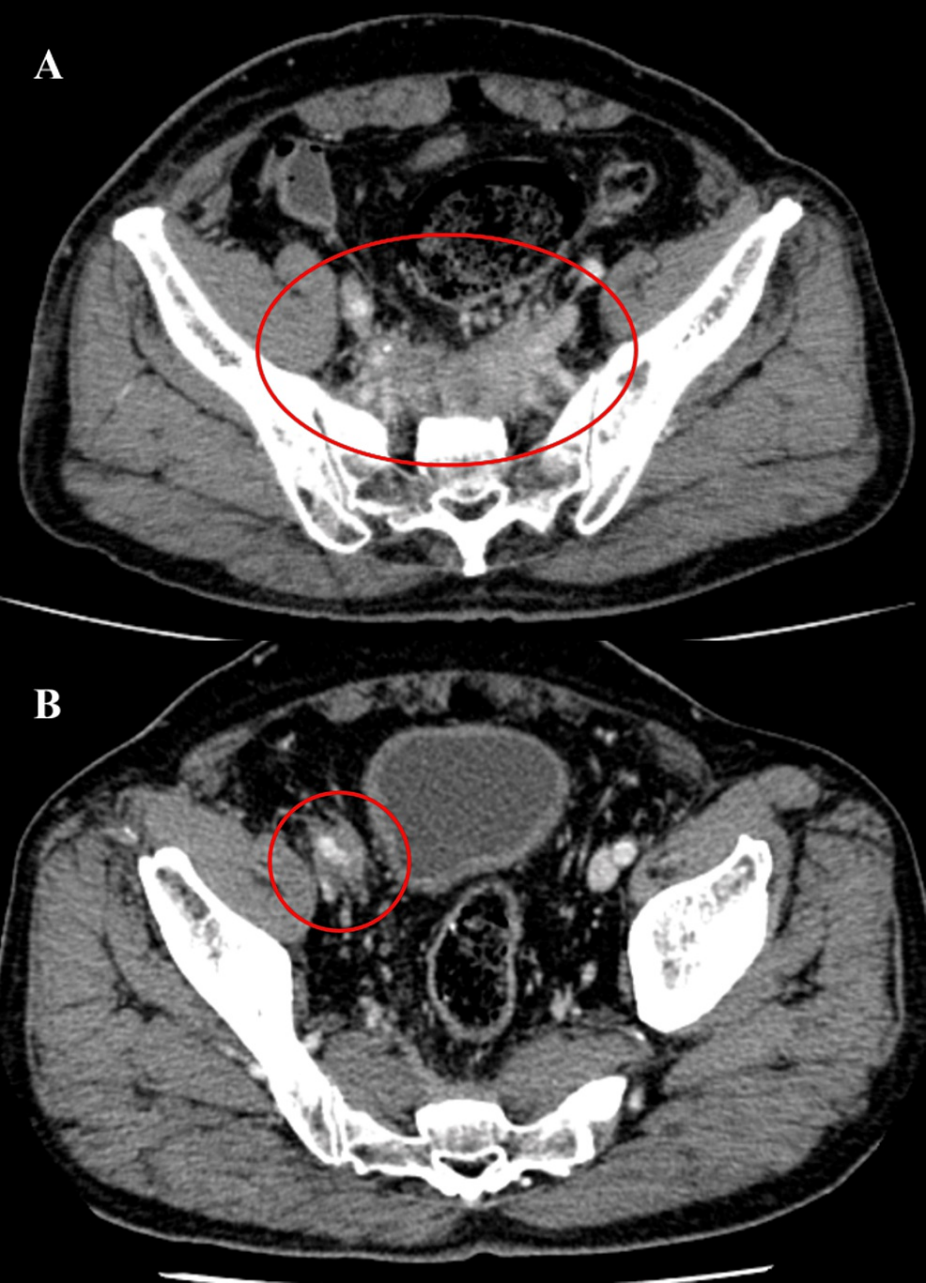
Contrast-enhanced abdominal computed tomography Contrast-enhanced abdominal computed tomography revealed soft-tissue shadows around the ventral sacrum (A) and right external iliac artery and vein (B).

The patient was conscious and had no abnormal vital signs. Physical examination revealed pitting edema in both lower extremities, predominantly in the right lower leg, no jugular vein distention, and no abnormal findings in terms of breathing, circulation, digestive organs, muscles, or joints. Blood investigations on admission are shown in Table 1. Blood investigations revealed normal liver and kidney functions, lactate dehydrogenase, inflammatory markers, complete blood count, and no eosinophilia. Immunological tests were negative for rheumatoid factor, myeloperoxidase, and proteinase 3 antineutrophil cytoplasmic antibody and positive for antinuclear antibody (×160, homogeneous). Contrast-enhanced abdominal CT revealed soft-tissue shadows around the ventral sacrum and right external iliac artery and vein; however, the soft-tissue shadows were not accompanied by invasion into the surrounding bone or muscle (Figure 1).

**Table 1:** Blood investigations on admission AST, aspartate aminotransferase; ALT, alanine aminotransferase; γGTP, γ-glutamyl transferase; LD, lactate dehydrogenase; ALP, alkaline phosphatase; BUN, blood urea nitrogen; WBC, white blood cell; RBC, red blood cell; MCV, mean corpuscular volume; IgG4, immunoglobulin G4; MPO-ANCA, myeloperoxidase anti-neutrophil cytoplasmic antibody; PR3-ANCA, proteinase-3-antineutrophil cytoplasmic antibody; ANA, antinuclear antibody; TSH, thyroid-stimulating hormone; BNP, brain natriuretic hormone; PT, prothrombin time; APTT, activated partial thromboplastin time; CEA, carcinoembryonic antigen; CA19-9, carbohydrate antigen 19-9; sIL-2R, soluble interleukin-2 receptor; HBs Ag, hepatitis B surface antigen; HBs Ab, hepatitis B surface antibody; HBV-DNA, hepatitis B virus deoxyribonucleic acid; HCV Ab, hepatitis C virus antibody; INF-ɤ, interferon-ɤ

Blood investigations on admission		
Biochemical examination		Range
Total protein, g/dL	6.8	6.5-8.2
Albumin, g/dL	3.9	3.7-5.5
AST, IU/L	16	10-40
ALT, IU/L	12	5-45
γGTP, IU/L	15	0-79
LD, IU/L	171	124-222
ALP, IU/L	96	38-113
Amylase, U/L	50	39-113
Creatine kinase, IU/L	67	50-230
BUN, mg/dL	20.6	8-20
Creatinine, mg/dL	0.83	0.65-1.09
Sodium, mEq/L	141	135-145
Potassium, mEq/L	4.0	3.5-5.0
Chlorine, mEq/L	105	98-108
Glucose, mg/dL	159	70-109
Hemoglobin A1c, %	6.1	4.6-6.2
Complete blood cell count		
WBC, /µL	5500	3500-9700
Neutrophil count, /µL	3550	-
Basophil count, /µL	50	-
Eosinophil count, /µL	230	-
Lymphocyte count, /µL	1230	-
Monocyte count, /µL	450	-
RBC, /µL	367 × 10^4^	438-577
Hemoglobin, g/dL	11.1	13.6-18.3
MCV, fL	91	83-101
Platelet, /µL	17.3 × 10^4^	14.0-37.9
Immunoserological examination		
C-reactive protein, mg/dL	1.08	<0.30
IgG4, mg/dL	102	<121
MPO-ANCA, IU/mL	0.2	<3.5
PR3-ANCA, IU/mL	0.6	<2.0
ANA	1:160	1:40
Rheumatoid factor, IU/mL	2	<15
Endocrinological examination		
TSH, µIU/mL	1.98	0.5-5.0
Free T_4_, ng/dL	1.34	0.9-1.7
BNP, pg/mL	40.5	<18.4
Coagulation examination		
PT, %	77.8	70-140
APTT, second	23.8	26.0-38.0
D-dimer, µg/mL	2.6	<1.0
Tumor marker		
CEA, ng/mL	1.9	<5.0
CA19-9, U/mL	3.4	<37
sIL-2R, U/mL	1100	122-496
Infection disease examination		
HBs Ag, IU/mL	0.33	<0.05
HBs Ab, mIU/mL	<10	<10
HBV-DNA, Log IU/mL	<1.0	<1.0
HCV Ab, S/CO	0.1	<0.9
IFN-ɤ	-	-
β-D-glucan, pg/mL	4.8	<20

**Figure 2:**
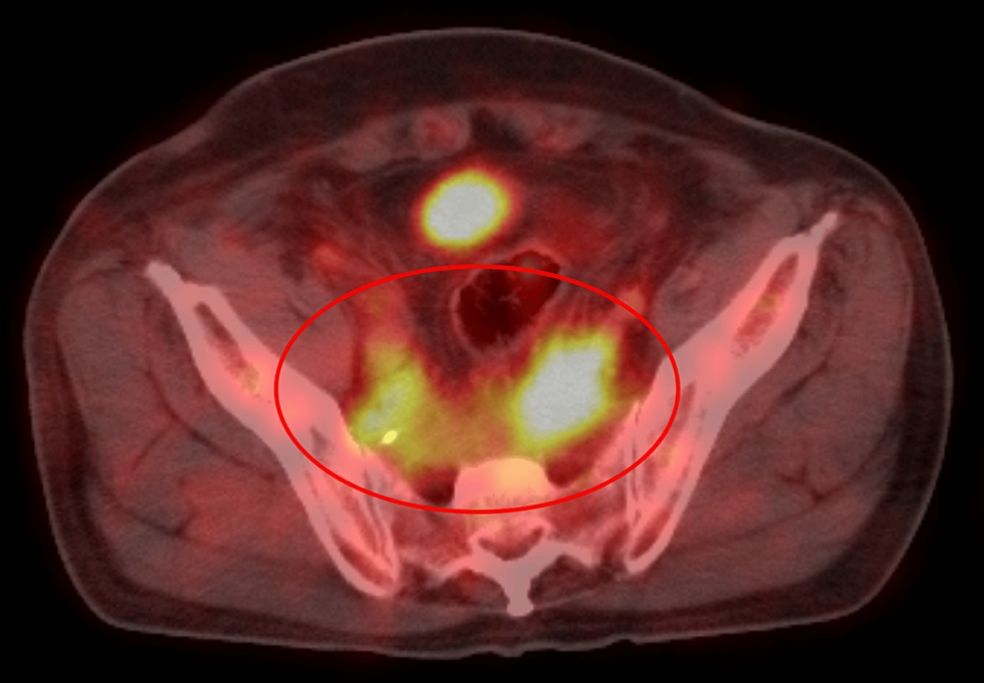
Positron emission tomography-computed tomography Positron emission tomography-computed tomography showed a significant accumulation of fluorodeoxyglucose with a standardized uptake value of 4-5 at the ventral sacrum and right external iliac artery and vein.

We considered the following differential diagnoses: colon cancer recurrence, malignant lymphoma, infectious abscess, and idiopathic, secondary, or IgG4-RD RPF [3]. CEA and CA19-9 levels were within the standard ranges for evaluation of colon cancer recurrence. Five months earlier, his serum sIL-2R had been 569 U/mL (reference: 122-496 U/mL), but it had increased to 1,100 U/mL at this visit. PET-CT revealed a significant accumulation of fluorodeoxyglucose, with a standardized uptake value (SUV) of 4-5 at the same sites as the soft-tissue shadows but no accumulation in other organs (Figure 2). This value was lower than the SUV of 10-13 at the time of diagnosis of transverse colon cancer two years earlier. Infectious diseases, such as bacterial infections, tuberculosis, and fungal infections, were investigated, but blood culture tests and interferon-gamma release assays were negative, and β-D glucan was 4.8 pg/mL (reference: <20 pg/mL). The patient had positive antinuclear antibody with a homogeneous pattern but no typical findings of systemic lupus erythematosus. Serum IgG4 was 102 mg/dL (reference: 11-121 mg/dL). We were unable to reach a diagnosis for the patient based on these indirect examinations during our initial evaluation.

**Figure 3:**
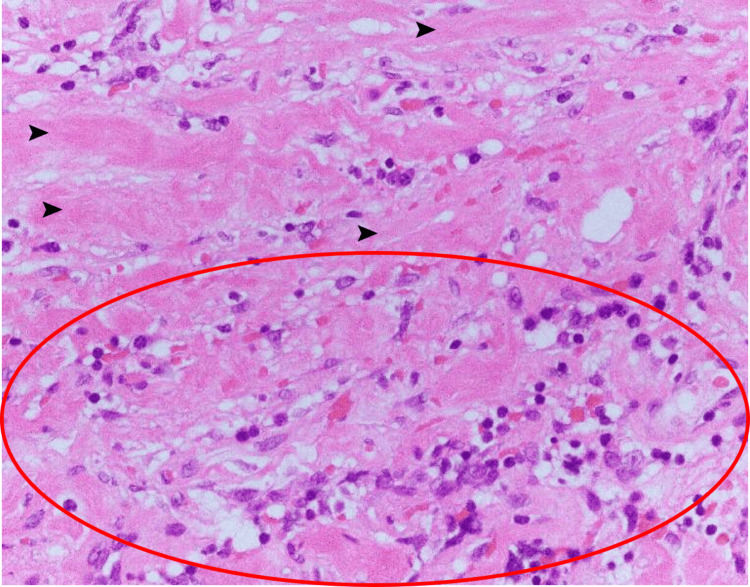
Hematoxylin and eosin staining Hematoxylin and eosin staining showed marked lymphocyte and plasma cell infiltration (〇) and fibrosis (➤) but no evidence of colon cancer metastasis or malignant lymphoma.

**Figure 4:**
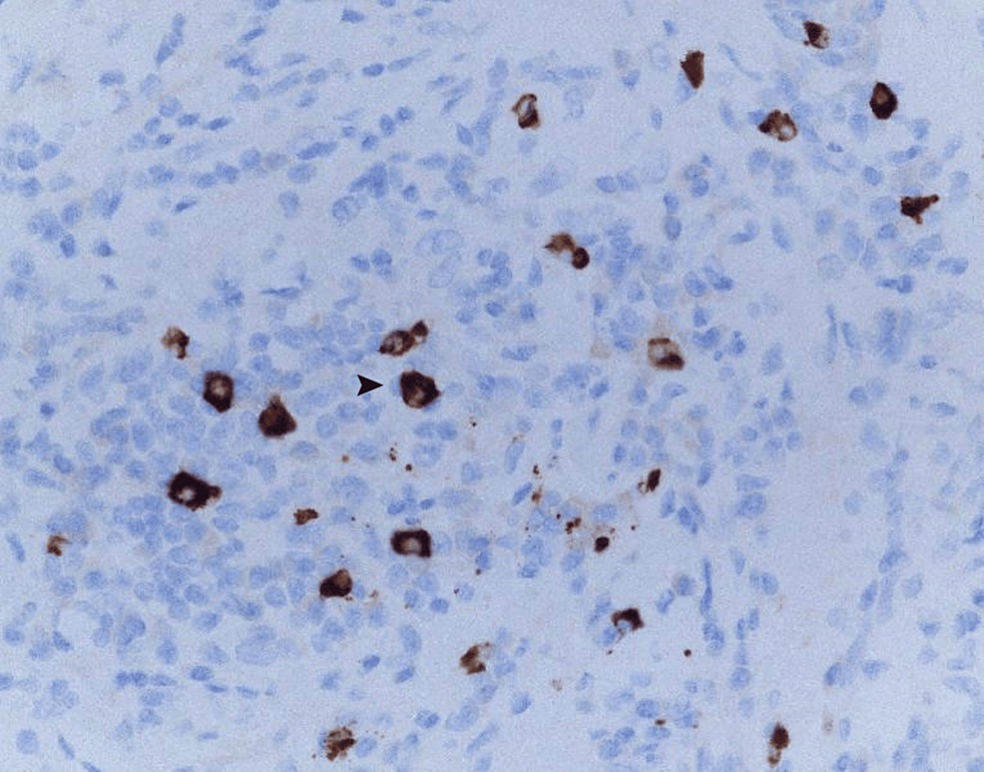
Immunoglobulin G4 staining Immunoglobulin G4 (IgG4) staining findings showed marked IgG4-positive cell infiltration (➤), with an IgG4-positive/IgG-positive cell ratio of 65% (reference: <40%) and infiltration of IgG4-positive plasma cells at >10 cells per high-power field.

We, therefore, performed laparotomy histology for diagnosis. Histopathological findings using hematoxylin and eosin staining showed marked lymphocyte and plasma cell infiltration and fibrosis but no evidence of colon cancer metastasis or malignant lymphoma (Figure 3). IgG4 staining showed marked IgG4-positive cell infiltration (Figure 4), with an IgG4-positive/IgG-positive cell ratio of 65% (reference: <40%) and infiltration of IgG4-positive plasma cells at >10 cells per high-power field. We ultimately diagnosed the patient with IgG4-RD RPF [11]. Although the patient did not have serologically high IgG4 (>135 mg/dL) according to the revised 2020 comprehensive diagnostic criteria for IgG4-RD, we also diagnosed the patient with probable IgG4-RD RPF based on imaging and pathological findings [12].

**Figure 5:**
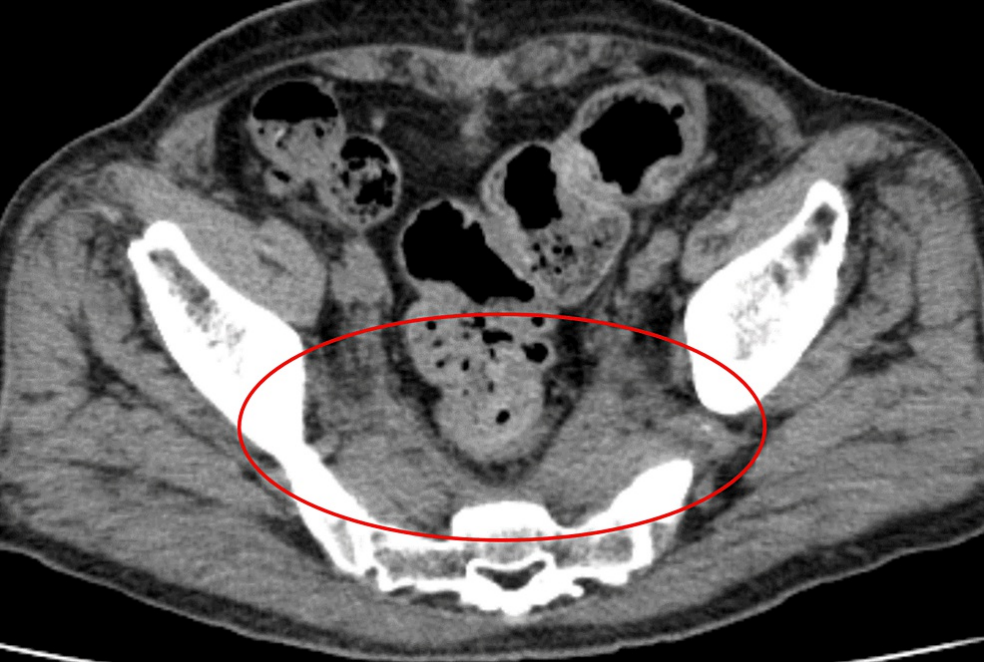
Abdominal computed tomography approximately 1 month after the start of treatment Abdominal computed tomography showed shrinkage of the soft-tissue masses around the ventral sacrum.

The patient was treated with prednisolone 30 mg/day (0.6 mg/kg). Approximately one month later, his lower leg edema had subsided, and abdominal CT showed shrinkage of the soft-tissue masses (Figure 5). He was subsequently discharged and followed up as an outpatient. During a two-month follow-up, there was no recurrence of his symptoms, his serum IgG4 level decreased to 19 mg/dL, and his sIL-2R level reduced to 527 U/mL. He showed improvements in terms of clinical, radiological, and blood parameters.

## Discussion

Here, we report a patient who developed IgG4-RD RPF after transverse colon cancer surgery but had normal IgG4 levels. In this case, the absence of elevated blood levels of generic tumor markers and IgG4 levels meant that the diagnosis could not be confirmed without a biopsy. The following points helped to confirm the diagnosis: elevated sIL-2R levels, no invasion of RPF mass to surrounding organs on imaging, and lower SUV on PET-CT compared with cancer. Together with these combined findings, the biopsy of RPF mass could be positive for IgG4-RD.

In the case of a patient with RPF, it is necessary to distinguish between idiopathic, secondary, and IgG4-RD because the treatment strategies differ depending on the cause [3]. The current patient had a history of colon cancer, and we therefore first considered cancer recurrence or RPF secondary to cancer. We also considered IgG4-RD because previous studies reported that patients with a history of malignancy were 2.5-4.5 times more likely to develop IgG4-RD than those without such a history [4,5]. Malignancies produce CD4+ cytotoxic T lymphocytes, which are involved in the development of IgG4-RD [13], and the T cells can persist after treatment of malignancy [14]. Notably, malignancy may occur not only before but also after the diagnosis of IgG4-RD [6]. Furthermore, Wallace et al. reported that IgG4-RD did not occur at the site of a previous malignancy [4], suggesting that the development of IgG4-RD RPF after a history of cancer is immune-mediated rather than caused by local changes associated with the malignancy. Clinicians should thus consider the association between malignancy and IgG4-RD when encountering patients with RPF with a history of malignancy.

Elevated serum IgG4 levels are typically relevant for diagnosing IgG4-RD; however, about 30% of patients with IgG4-RD have normal IgG4 levels despite the presence of IgG4 in histopathological and immunohistochemical findings [1]. Furthermore, IgG4 levels do not necessarily reflect the disease activity of IgG4-RD accurately [15]. In these cases, serum sIL-2R levels reflect the inflammatory process associated with increased T-cell activation and are thus a useful diagnostic marker for IgG4-RD [7,8]. Serum sIL-2R levels have also been reported to be valuable for assessing disease activity, the number of affected organs, therapeutic response, and relapse of IgG4-RD [16-18]. sIL-2R can thus be considered a useful diagnostic tool for IgG4-RD.

IgG4-RD RPF is generally difficult to differentiate from malignancy [3]. Imaging techniques may help to diagnose IgG4-RD RPF and differentiate it from malignant RPF, with anterior displacement of the aorta by RPF on CT suggesting the possibility of malignancy [19]. Although not used in the present case, the magnetic resonance imaging (MRI) feature of RPF secondary to cancer is described as the destruction or invasion of adjacent muscle and bone structures [20]. Notably, the SUV in PET-CT is lower for IgG4-RD than for cancer [9,10]; indeed, the SUV of IgG4-RD RPF in this case was 4-5, compared with an SUV of 13 at the time of diagnosis of transverse colon cancer. Imaging findings from CT, MRI, and SUV on PET-CT may thus also be useful diagnostic tools for IgG4-RD.

## Conclusions

We report the case of a patient in whom IgG4-RD RPF was the cause of isolated RPF that developed after transverse colon cancer surgery. IgG4-RD RPF should be considered when isolated RPF appears in a patient with a history of colorectal cancer showing normal IgG4 levels. The combined findings of serum sIL-2R, surrounding mass imaging, and/or SUV PET-CT might prompt a biopsy of the mass, which can aid the diagnosis of IgG4-RD RPF. This case thus highlights such a helpful diagnostic approach to the combined findings of sIL-2R, surrounding mass images, and SUV PET-CT in IgG4-RD RPF in patients with normal IgG4 levels after colorectal cancer surgery.
